# InferBERT: A Transformer-Based Causal Inference Framework for Enhancing Pharmacovigilance

**DOI:** 10.3389/frai.2021.659622

**Published:** 2021-05-26

**Authors:** Xingqiao Wang, Xiaowei Xu, Weida Tong, Ruth Roberts, Zhichao Liu

**Affiliations:** ^1^ Department of Information Science, University of Arkansas at Little Rock, Little Rock, AR, United States; ^2^ FDA/National Center for Toxicological Research, Jefferson, AR, United States; ^3^ ApconiX Ltd, Alderley Park, Alderley Edge, United Kingdom; ^4^ Department of Biosciences, University of Birmingham, Birmingham, United Kingdom

**Keywords:** artificial intelligence, natural language processing, language models, causal inference, pharmacovigilance

## Abstract

**Background:** T ransformer-based language models have delivered clear improvements in a wide range of natural language processing (NLP) tasks. However, those models have a significant limitation; specifically, they cannot infer causality, a prerequisite for deployment in pharmacovigilance, and health care. Therefore, these transformer-based language models should be developed to infer causality to address the key question of the cause of a clinical outcome.

**Results:** In this study, we propose an innovative causal inference model–InferBERT, by integrating the A Lite Bidirectional Encoder Representations from Transformers (ALBERT) and Judea Pearl’s Do-calculus to establish potential causality in pharmacovigilance. Two FDA Adverse Event Reporting System case studies, including Analgesics-related acute liver failure and Tramadol-related mortalities, were employed to evaluate the proposed InferBERT model. The InferBERT model yielded accuracies of 0.78 and 0.95 for identifying Analgesics-related acute liver failure and Tramadol-related death cases, respectively. Meanwhile, the inferred causes of the two clinical outcomes, (i.e. acute liver failure and death) were highly consistent with clinical knowledge. Furthermore, inferred causes were organized into a causal tree using the proposed recursive do-calculus algorithm to improve the model’s understanding of causality. Moreover, the high reproducibility of the proposed InferBERT model was demonstrated by a robustness assessment.

**Conclusion:** The empirical results demonstrated that the proposed InferBERT approach is able to both predict clinical events and to infer their causes. Overall, the proposed InferBERT model is a promising approach to establish causal effects behind text-based observational data to enhance our understanding of intrinsic causality.

**Availability and implementation:** The InferBERT model and preprocessed FAERS data sets are available on GitHub at https://github.com/XingqiaoWang/DeepCausalPV-master.

## Introduction

The rise of artificial intelligence (AI) has transformed many aspects of human life, especially in healthcare, personal transport, law-making, and entertainment (Silver et al., 2017; Awad et al., 2018; Topol, 2019; Woo, 2019). One of the breakthroughs in AI is the advent of transformer-based language models, that can achieve state-of-the-art (SOTA) performance in a wide range of natural language processing (NLP) tasks (Devlin et al., 2018; Lan et al., 2019; Brown et al., 2020; Zaheer et al., 2020). Data set size and the number of parameters tend to increase exponentially with language model development in pursuit of improved model performance. For example, the GPT3 model consisted of 175 billion parameters and was trained with 499 billion tokens (Brown et al., 2020). Consequently, the achieved high prediction performance came at the expense of model interpretability (Moraffah et al., 2020). Another critical limitation of transformer-based language models is the lack of ability to infer causality. Model interpretability and lack of causal inference affect the dissemination of AI-powered models in critical fields, particularly in healthcare and pharmacovigilance where model interpretability is vital for deployment (Feder et al., 2020).

The goal of this project is to develop a model that can infer the causality of clinical outcome from unstructured pharmacovigilance reports. Causality (also referred to as causation or cause and effect) is the influence by which one event, process, or state (a cause) contributes to the production of another event, process or state (an effect). Causal inference is the process of identifying the cause and effect based on the conditions of the occurrence of the event (Pearl, 2010). There is a fundamental difference between causal inference and association inference: causal inference analyzes the response of the effect variable when the cause is changed (Pearl and Mackenzie, 2018).

One of the conventional approaches to prove cause and effect is a randomized controlled trial. In a randomized controlled trial, the test subject is randomly assigned to the treatment or control groups, which are identical in every way other than one group receives drug (treatment) and one receives placebo (control). If the clinical outcome is better in one group than the other with statistical significance, then causality is established. However, conducting a randomized controlled trial to establish causality relationships is often time consuming, expensive and can be impractical in the real world. For example, it would be impractical to conduct a randomized controlled trial to demonstrate causality regarding the impact of a vegetarian diet on life expectancy. Thus, there is a pressing need to develop AI-powered language models that can identify potential causality from accumulated real-world data.

Only one attempt has been made so far to perform causal inference using text as a potential cause of an effect (Veitch et al., 2020). The study proposed a low-level text representation (called causally sufficient embeddings) for empirical estimations of causal effects on observed text documents. Two text corpora were used to address the following specific causal questions: 1) Does adding a theorem to a paper affect its chance of acceptance? 2) Does labeling a post with the author’s gender affect post popularity? However, the approach required external treatment and outcome information for the text corpus and could not estimate the causal relationship among the variables within the text corpus. To date, there remains an absence of any reports that infer cause and effect relationships between terms or variables, (e.g. treatment and clinical outcome) within a text.

One of the potential applications of transformer-based language models for causal inference is pharmacovigilance. Pharmacovigilance, also known as drug safety, is the pharmacological science related to collecting, detecting, assessing, monitoring, and preventing adverse effects with pharmaceutical products (Edwards, 2012). The FDA Adverse Event Reporting System (FAERS) is an essential pharmacovigilance resource containing rich information on adverse event and medication error reports. The larger number of FAERS case reports comprising confounders, treatments, and clinical outcomes could be utilized to recognize adverse drug reactions (ADRs) and establish a potential causal relationship between the drug and the adverse events to further support regulatory decision making.

In this study, we propose a novel transformer-based causal inference model—InferBERT, by integrating A Lite Bidirectional Encoder Representations from Transformers (ALBERT) Lan et al. (2019) and Judea Pearl’s do-calculus Pearl (2010) to infer causality for pharmacovigilance using FAERS case report data. We employed two FAERS case report data sets to estimate the potential causes of Analgesics-related acute liver failure and Tramadol-related mortalities to prove the concept. Furthermore, identified causes were visualized by a proposed causal tree, which was calculated using recursive do-calculus and verified with evidence from clinical trial studies and FDA drug labeling.

## Materials and Methods

**FIGURE 1:**
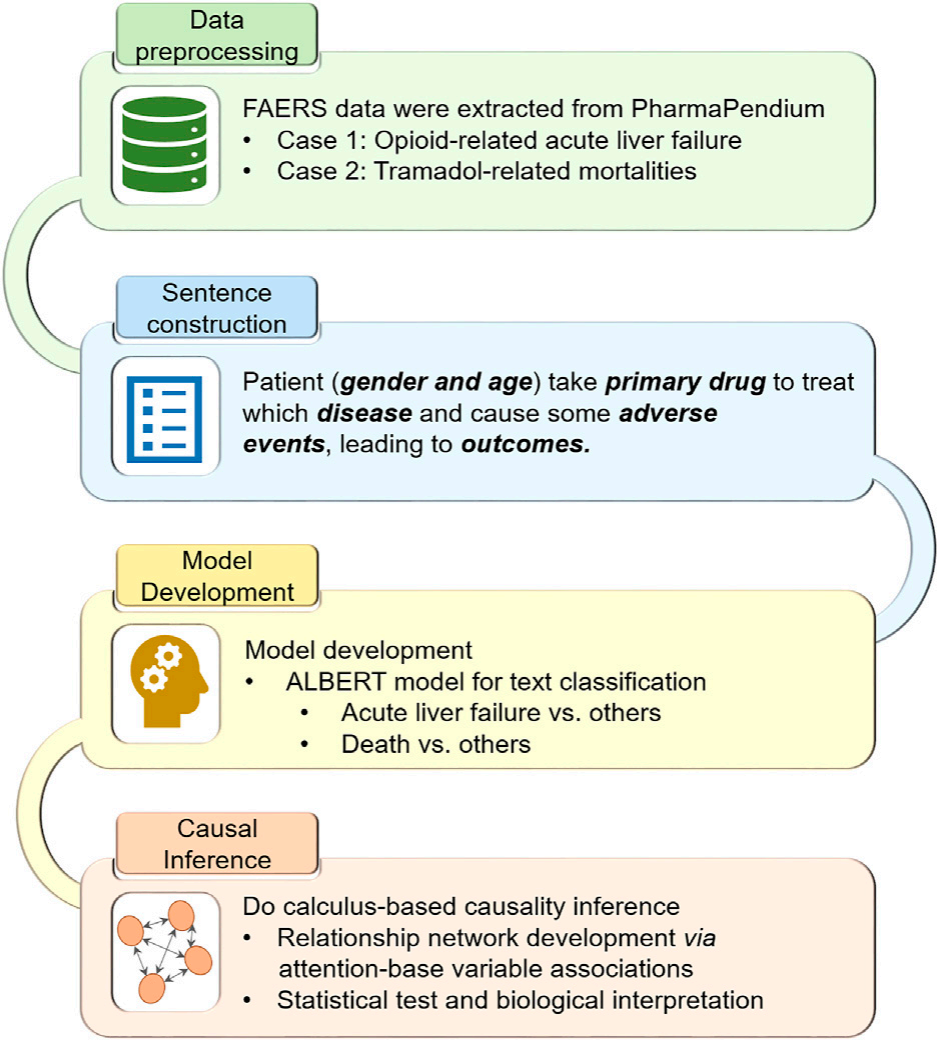
Workflow of the study.

Figure 1 illustrates the workflow of the study:

1. The FDA Adverse Event Reporting System (FAERS) case reports, including Analgesics-related acute liver failure and Tramadol-related mortalities, were extracted and preprocessed.
2. The preprocessed case reports were converted into the sentence-like descriptions for the subsequent pretrained language model ALBERT.
3. We fine-tuned the pretrained ALBERT model based on the transformed sentence-like descriptions to predict Analgesics-related acute liver failure and Tramadol-related mortalities, respectively.
4. Do-calculus was implemented into the fine-tuned ALBERT models for causal inference.

### Clinical Knowledge

The two critical aspects of causal relations in pharmacovigilance are 1) a drug causes the particular adverse drug reaction and 2) the causal relationship between the adverse drug reaction and different clinical factors needs to be established. Therefore, we employed two FAERS datasets, including Analgesics-induced acute liver failure, and Tramadol-related mortalities, to investigate the performance of the proposed Deep Causal Pharmacovigilance (InferBERT) approach.

#### Analgesics-Induced Acute Liver Failure

Analgesics or painkillers form a group of drugs used to achieve analgesia and relief from pain. Analgesics include acetaminophen (APAP), the nonsteroidal anti-inflammatory drugs (NSAIDs) such as the salicylates, and opioid drugs such as morphine and oxycodone. Analgesics are one of the most common causes of drug-induced acute liver failure (Björnsson, 2010). Among different analgesics, APAP-induced hepatotoxicity remains a global issue. For example, in the United States, it accounts for more than 50% of overdose-related acute liver failure (ALF), and approximately 20% of the liver transplant cases (Bernal and Wendon, 2013). Furthermore, APAP is also combined with prescribed—or is formulated with—opioid analgesics to boost pain relief, which increases the possibility of overdose or even abuse (Basco et al., 2016). The mortality rate of ALF is approximately 67–75% before liver transplantation (Bernal and Wendon, 2013). Also, it was reported that APAP-induced ALF is more common and more severe in women based on the Acute Liver Failure Study Group cohort study (Rubin et al., 2018).

#### Tramadol-Related Mortalities

Tramadol is an opioid-related medicine used to treat severe pain. In the United States, there is a Boxed Warning to Tramadol labeling to ensure appropriate inclusion of the serious adverse reactions such as addiction, abuse, and misuse, life-threatening respiratory depression, accidental ingestion, and interaction with drugs affecting cytochrome P450 isoenzymes. In particular, the statement “Do not prescribe tramadol for patients who are suicidal or addiction-prone. Consideration should be given to the use of non-narcotic analgesics in patients who are suicidal or depressed” is highlighted in the Drug Abuse and Dependence section of the US FDA label (http://dailymed.nlm.nih.gov/dailymed/downloadpdffile.cfm?setId=5bee381f-b14a-e62b-e053-2991aa0a3c2b). Furthermore, post-marketing adverse events such as QT prolongation and Torsade de Pointes have been reported with tramadol use, which is included in the Adverse Reaction section.

### Preprocessing of FAERS Case Reports

The FAERS case reports curated in the PharmaPendium database (https://www.pharmapendium.com/login/email) were used in this study. Specifically, we used the search query “Effects: (Acute liver fibrosis and cirrhosis, OR Acute liver failure and associated disorders, OR Cholestasis and jaundice) AND Drugs by AND-groups: [Analgesics (Any Role)]” to extract 45,773 FAERS case reports for Analgesics-induced acute liver failure. We employed the search query “Drugs: (Tramadol Hydrochloride) AND Drugs Reported Role: (Drug’s Reported Role: Primary Suspect Drug OR Secondary Suspect Drug)” and obtained 39,930 FAERS case reports for Tramadol-related mortalities.

The FAERS data in the PharmaPendium database has been preprocessed, including removing duplicating records, normalizing drug names, and standardizing adverse events terminology. However, some hurdles still exist for consolidating the information to carry out causal inference. Therefore, we implemented the following data cleaning procedure to further process the datasets:

1) We normalized the terms such as “UNK,” “UNKNOWN,” “()” and considered them as missing values.
2) Considering the different doses used in FAERS case reports, we unified the dose unit into milligram (mg). We categorized the dose into two classes: large than 100 mg and less than 100 mg.
3) We categorized the patient age into four groups: less than 18 years old, 18–39 years old, 40–64 years old, and older than 65 years.
4) For the tramadol-related mortalities dataset, we excluded the case reports without clinical outcome information since we used the clinical outcome as the prediction endpoint. As a result, we obtained a total of 36,661 and 27,245 case reports for Analgesics-induced acute liver failure and Tramadol-related mortalities, respectively.

### Sentence Generation With FAERS Case Reports

Our proposed model for causal inference, InferBERT, is based on the transformer model Lan et al. (2019), which is a sequence transduction model that requires sequences as the input. Therefore, we extracted sentences from each of the FAERS case reports. Specifically, the FAERS case reports are denoted as *D*, *D* = (*d*_1_, *d*_2_, … , *d*_N_), *d*_*i*_ is the *i*th case report of the dataset, *N* is the total number of case reports. Suppose that there are *M* clinical features, (e.g. gender, age, primary suspect drug, dose) for the FAERS case report dataset *D*. Each clinical feature consists of a set of terms. For example, the *jth* feature *f*_*j*_ consists of a set of terms *T*_*j*_ (e.g., feature gender includes terms male and female) as value, where *T*_*j*_ = (*t*_*j*1_, *t*_*j*2_, … , *t*_*jG*_), *G* represents the total number of terms for a particular clinical feature. For example, clinic feature “Indication” may take a value such as “Pain” or “Suicide Attempt.” Then, *d*_*i*_ = (*f*_*i*1_, *f*_*i*2_, … , *f*_*i*M_), where *f*_*ij*_ is the *jth* feature of the *ith* instance, and *f*_*ij*_ ⊂ *T*_j_. Without losing generality, we set the *f*_*m*_ as the end point, which means the *mth* clinical feature in the dataset *D* will be the target of classification and causal inference.

Then, we transformed each case report *d*_*i*_ into the corresponding sentence *s*_*i*_. For example, in the FAERS dataset, the clinical features included gender, age, primary suspect drug, dose, indication, adverse events, and outcomes in each case report d_i_. The generated sentence followed the template listed below:

Patient (gender and age) takes a primary suspect drug to treat which disease and cause some adverse events, leading to outcomes.

Then we generated the sentence set *S*, *S* = (*s*_1_, *s*_2_, … , *s*_*N*_).

For the Analgesics-induced acute liver failure data, the term “acute liver failure” in clinical feature “adverse event” was used as the endpoint. Of 36,661 FAERS case reports, 15,224 cases with “acute liver failure” were considered as positives and remaining 21,437 cases as negatives (positive/negative ratio = 0.71). For Tramadol-related death data, the clinical feature “outcomes” was used as the endpoint. The case reports with the term “death” in the clinical feature “outcomes” were considered as positives and other case reports were used as negatives. Accordingly, a total of the 27,245 case reports with 9,846 positives and 17,399 negatives were obtained (positive/negative ratio = 0.57). Next, we employed a stratified splitting strategy to divide each sentence set *S* into three sets, including a training set (for training the model), a development set (for model selection), and a test set (for model validation) with an approximate ratio of 0.64: 0.16: 0.20. The detailed information of the two datasets was listed in Table 1.

**TABLE 1:** Sentence sets of Analgesics-related acute liver failure and Tramadol-related mortalities.

Endpoints	Datasets	Number of positives	Number of negatives	Positive versus negative ratio
Acute liver failure	Total	15,224	21,437	0.71
	Training set	9,798	13,663	0.71
	Develop set	2,399	3,467	0.69
	Test set	3,027	4,307	0.70
Tramadol-related death	Total	9,846	17,399	0.57
	Training set	6,250	11,185	0.56
	Develop set	1,588	2,722	0.57
	Test set	2,008	3,442	0.58

### ALBERT-Based Classification Model

Bidirectional Encoder Representations from Transformers (BERT) is a transformer that learns contextual bidirectional representations from unlabeled text documents by jointly conditioning on both left and right contexts (Vaswani et al., 2017; Devlin et al., 2018). BERT employed two training strategies, including a masked language model (MLM) and Next Sentence Prediction (NSP), to learn bidirectional representations. In the MLM, 15% of words in a sequence are replaced with a (MASK) token, and the model attempts to predict the original value of the masked words, based on the context provided by the other, non-masked, words in the sequence. In the NSP, the model receives pairs of sentences as input and learns to predict if the second sentence in the pair is the subsequent sentence in the original document. The BERT model has achieved state-of-the-art performance on most NLP tasks, requiring minimal task-specific architectural modification.

Increasing the model size of pre-trained language models often results in an improved model performance for downstream tasks. However, The GPU/TPU memory limitations, longer training times, and model overfitting generate obstacles to further expand the model size. To address these obstacles, Google AI proposed a Lite BERT (ALBERT) by adopting three techniques to trim down BERT (Lan et al., 2019). First, factorized embedding parameterization was used to break down token embeddings into two small embedding matrixes. After applying this decomposition, embeddings parameters can be reduced from (number of tokens × hidden layer size) to (number of tokens × token embedding size + token embedding size × hidden layer size). The reduction of parameters is significant, especially when the hidden layer size is much larger than the token embedding size. Second, cross-layer parameter sharing was proposed to prevent an increasing number of parameters with increased depth of the model. ALBERT is configured to share all parameters which include feed-forward network and attention parameters across layers. Lastly, a sentence-order prediction (SOP) loss was developed to model inter-sentence coherence in ALBERT, enabling the new model to perform more robustly in multi-sentence encoding tasks. To summarize, we chose ALBERT over BERT because it achieved an equivalent accuracy, if not better, with a much smaller model size.

The ALBERT_base_ classification model was employed to classify the endpoint term of each instance. We build a simple SoftMax classifier for the downstream classification task of the ALBERT model. In the ALBERT model, the learned representation vector of the (CLS) special token of the last layer acts as the input of the downstream model, with no hidden layers. The dimensionality of the output layer in the classification model is two, where the SoftMax function is adopted to classify whether the endpoint term exists or not. The loss function of the classification model is shown as follows:

$$CE=-\sum_{i}^{N}p_{i}*\log\left(F\left(s_{i}\right)\right)$$ (1)

where, *F*(*s*_*i*_) is the output of the classification model for *s*_*i*_, which is a calculated probability of the predicted class of *s*_*i*_. *p*_*i*_ is the true probability of the end point of *s*_*i*_.

We denote *p*′_*i*_ = *F*(*s*_*i*_), as the output of classification model, where *p*′_*i*_ is the positive probability of the end point for instance *i*. Then, the output set, (i.e. conditional probability distribution) of the classification model can be denoted as *O*, *O* = ( *p*′_1_, *p*′_2_, … , *p*′_N_), *i* ϵ (1, 2, … , *N*).

### Causal Inference Using Do-Calculus Section

Since the transformer is a generative model, the ALBERT based classification model can be seen as a conditional probability distribution *p* (endpoint|clinical features) of the endpoint in the clinical feature in FAERS case reports. However, this conditional probability distribution could not provide convincing evidence of causal effects, similar in the way as one cannot conclude causal effects from a randomized clinical trial with only the treatment group. To empirically estimate the potential clinical features causing the endpoint, we used the Judea Pearl’s Do-calculus framework (Tucci, 2013; Pearl and Mackenzie, 2018). The Do-calculus aims to investigate the interventional conditional probability distribution of *p*[endpoint|DO(clinical features)] by counterfactually changing the clinical features. In this study, we considered the clinical features as the cause of the endpoint if there is a statistically significant difference between the interventional conditional probability distributions of *p*[endpoint|DO(clinical features)] and *p*[endpoint|NOT DO(clinical features)].

Based on the conditional probability distribution *O* generated from our developed ALBERT_base_ classifier, we performed the Do-calculus procedure to estimate the cause of the endpoint. The pseudo code of the Do-calculus procedure is shown below.

**Algorithm 1:** Do-calculus algorithm.


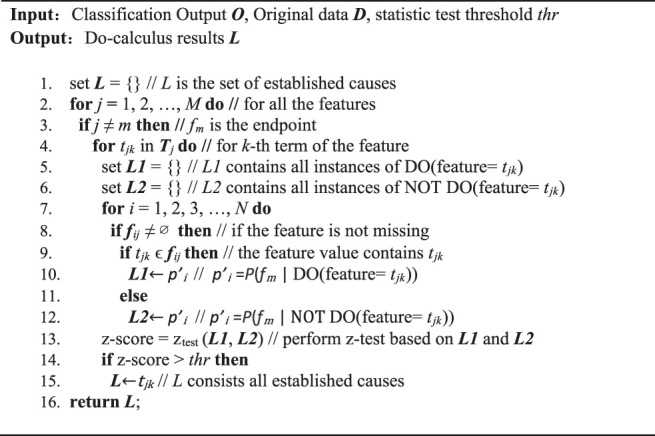


For all the terms in each clinical feature, we applied the Do-calculus algorithm to check whether it is the cause of the endpoint. For a term *t*_*jk*_, if a case report *d*_*i*_ contains *t*_*jk*_, we say it is Do *t*_*jk*_, while if *f*_*ij*_≠∅ and *t*_*jk*_ is not in *f*_*ij*_, then it is not do *t*_*jk*_. We assigned the case *d*_*i*_ to different sets, *L1* and *L2*. *L1* is the set of case reports do *t*_*jk*_, while *L2* consists of those case reports not do *t*_*jk*_. We used the one tail z-test to evaluate whether instances in *L1* have significant differences to those in *L2*. For example, if the endpoint term is *f*_*m*_ and we want to see the impact of *t*_*11*_ (the first term of the first feature), then for each instance *d*_*i*_ we have the probability of *f*_*m*_ being positive as follows:

$$p\left(f_{m}|f_{1},f_{2},\ldots ,f_{j},\ldots ,f_{M}\right),\;j\neq m.$$ (2)

As shown in Eqs. 3, 4, for those instances do *t*_11_, the set is *L1*, while those not do the set is *L2*.

$$L1=\left\{{p^{\prime }}_{i}\;|\;{p^{\prime }}_{i}=\;p\left(f_{m}|t_{11}\epsilon f_{i1},f_{i2},\ldots ,f_{ij},\ldots ,f_{iM}\right),\;j\neq m\right\},$$ (3)

$$L2=\left\{{p^{\prime }}_{i}\;|\;{p^{\prime }}_{i}=p\left(f_{m}|\;t_{11}\notin f_{i1},f_{i2},\ldots ,f_{ij},\ldots ,f_{iM}\right),\;j\neq m\right\}$$ (4)

To establish all the causal terms of the end point, we evaluated every term in each feature. This generated the term set *L*, which is the set of all the terms in each feature that satisfy the statistical significance test.

### Causal Tree Construction

To further explore the causal relationship among the enriched causal terms, we built a causal tree based on the Do-calculus. For each term in *L*, which has significant relationship with the end point *f*_*m*_, we explored the secondary causal terms. For example, if *t*_11_ is a term in *L* (i.e., an established cause), and we wanted to verify whether *t*_21_ is a secondary cause for the endpoint *f*_*m*_, then we fixed the *t*_11_ term and performed a statistical significance test on the difference between the instances following distribution shown as Eq. 5 and that following distribution shown as Eq. 6.

$$p\left(f_{m}|t_{11}\epsilon f_{1},t_{21}\epsilon f_{2},\ldots ,f_{j},\ldots ,f_{M}\right),\;j\neq m,$$ (5)

$$p\left(f_{m}|t_{11}\epsilon f_{1},\;t_{21}\notin f_{2},\ldots ,f_{j},\ldots ,f_{M}\right),\;j\neq m.$$ (6)

By recursively performing the do-calculus algorithm on the subset of *L*1, we found the set of secondary terms *L*′ based on each term *t* in the set *L*. Consequently, the enriched causal factors could be arranged in a hierarchical tree structure. To provide more interpretable results, we only focused on the most significant causal factors by restricting the maximum number of causal factors that is equal to the tree level. In other words, in tree level *N*, the number of retained causal factors was less or equal to *N*.

### Robustness Evaluation

The proposed InferBERT model is based on the fine-tuned pretrained ALBERT_base_ for text classification and causal inference. Application of pretrained language models to the supervised downstream task is designed in the BERT model and its derivatives such as ALBERT. However, this process can be less than robust: even with the same parameter values, distinct random seeds can lead to different results (Dodge et al., 2020). To investigate the reproducibility of our InferBERT model, we repeated parallel experiments with the same parameters. Two strategies were applied to compare the enriched causal terms (terms hereafter) from the do-calculus algorithm. First, a Venn diagram was used to compare the consistency of the enriched terms among the different runs. The average percentage of enriched terms per repeated run was calculated by the following Eq. 7:

$$\text{average percentage of enriched terms}=\frac{\sum_{i=1}^{T}\text{number of common enriched terms in all runs }\text{number of enriched terms in run }i\;}{T},$$ (7)

where *T* is total number of repeated runs.

Second, the percentage of overlapped terms (POT) strategy was used to investigate the consistency of the order of enriched terms. Specifically, we ranked the enriched terms based on their z-score from high to low. We then calculated the POT by the number of overlapping terms among three repeated runs divided by the number of enriched terms in each subset of the ranked enriched term list.

### Conventional Causal Inference Methods

To further verify the results yielded by the proposed InferBERT model, we employed three conventional causal inference methods including the proportional reporting ratio (PRR) Evans et al. (2001), the reporting odds ratio (ROR) Van Puijenbroek et al. (2002), and the empirical Bayes geometric mean (EBGM) Szarfman et al. (2002) for the causal inference of the two datasets. Specifically, we calculated the signal scores for the enriched teams from the proposed InferBERT model using the three conventional approaches to investigate whether these clinically verified terms could be identified. The three conventional methods are widely used for safety signal detection to prioritize the potential causal factors in FAERS datasets. PRR and ROR are based on the case frequency and statistical measures, while EBGM is based on Bayesian estimation. In this study, the standard cut-off values for enriching the safety signal were used. For the PRR, a signal is detected if the number of co-occurrences is three or more and the PRR is two or more with an associated χ^2^ value of four or more. For the ROR, a signal is detected if the lower limit of the 95% two-sided confidence interval exceeds one. For the EBGM, a signal is enriched when the lower one-sided 95% confidence limit of the EBGM (EB05) equal or more than two.

### Implementation of the InferBERT

To facilitate the application of our model, we developed a standalone package to simplify the implementation process. The current version of the InferBERT is based on a lite version of BERT (ALBERT, https://github.com/google-research/bert) under Python 3.6 and TensorFlow version 1.15. We evaluated our proposed InferBERT model on one NVIDIA Tesla V100 GPU. For Analgesics-induced acute liver failure and Tramadol-related mortalities datasets, the average runtime was 7.5 and 6 h. We incorporated the causal function into the ALBERT source code and make it publicly available through https://github.com/XingqiaoWang/DeepCausalPV-master.

## Results

### Construction of Artificial Sentences Based on FAERS Case Reports

**FIGURE 2:**
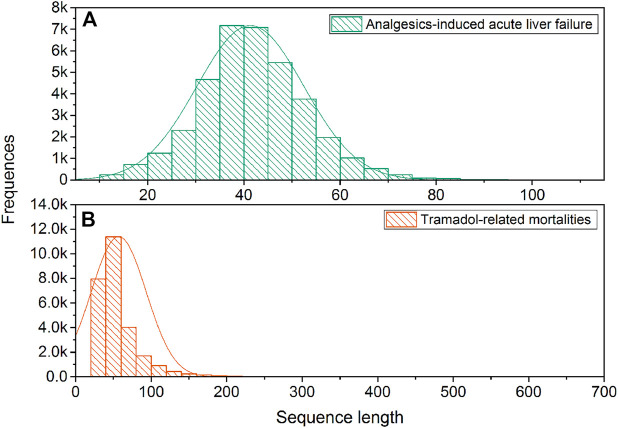
The distribution of sequence length: **(A)** Analgesics-induced acute liver failure; and **(B)** Tramadol-related mortalities.

Figure 2 illustrates the sequence length distribution for two sentence sets, respectively. The average and standard deviation of sequence lengths were 41.34 ± 11.14 and 56.94 ± 36.40 for the Analgesics-induced acute liver failure and Tramadol-related mortalities sentence sets, respectively. Considering the adverse event feature was designed as the endpoint for Analgesics-induced acute liver failure, the shorter average sequence was expected. We further calculated the term frequency-inverse document frequency (tf-idf), and the top 10 terms with the highest tf-idf values are listed in Table 2. The most frequent terms for Analgesics-induced acute liver failure sentence set were acetylcysteine, *acinetobacter*, alafenamide, altered, and appendicectomy. Terms including abacavir, indomethacin, glossodynia, idiopathic, and amnestic showed the highest tf-idf values in the Tramadol-related mortalities sentence set.

**TABLE 2:** Top 10 most frequent terms in the two sentence sets based on the tf-idf values.

Analgesics-related acute liver failure		Tramadol-related mortalities	
Terms	Tf-idf value	Terms	Tf-idf value
Acetylcysteine	0.0318	Abacavir	0.0323
Acinetobacter	0.0318	Indomethacin	0.0323
Alafenamide	0.0318	Glossodynia	0.0315
Altered	0.0318	Idiopathic	0.0315
Appendicectomy	0.0318	Amnestic	0.0312
Appetite	0.0318	Assault	0.0312
Assist	0.0318	Axetil	0.0312
Atherosclerosis	0.0318	Bradyarrhythmia	0.0312
Brucellosis	0.0318	Brugada	0.0312
Cabazitaxel	0.0318	Cardiorenal	0.0312

### ALBERT Model Development

ALBERT_base_ model developed on the 16G BOOKCORPUS Zhu et al. (2015) and English Wikipedia Devlin et al. (2018) were employed in this study. The ALBERT_base_ model consisted of 12 repeating layers, 128 embeddings, 768 hidden, and 12 heads with 11 M parameters. We further fine-tuned the ALBERT_base_ model with training sets and determined the optimized models based on text classification results in the development sets for the endpoints, (i.e. acute liver failure and death). We used one NVIDIA V100 (32 GB) GPU for fine-tuning the model. For the Analgesics-induced acute liver failure dataset, the maximum sequence length was fixed to 128, and the mini-batch size was set to 128. A total of 10,000 training steps were implemented with 2,000-step warmup, and the checkpoint step was set to 500 for recording the prediction results. For the Tramadol-related mortalities dataset, we used the same parameter settings except for a longer maximum sequence length, (i.e. 256). More training steps, (i.e. 20,000 steps) were selected as well since the Tramadol-average sequence length was longer than that of the Analgesics-induced acute liver failure dataset.

**FIGURE 3:**
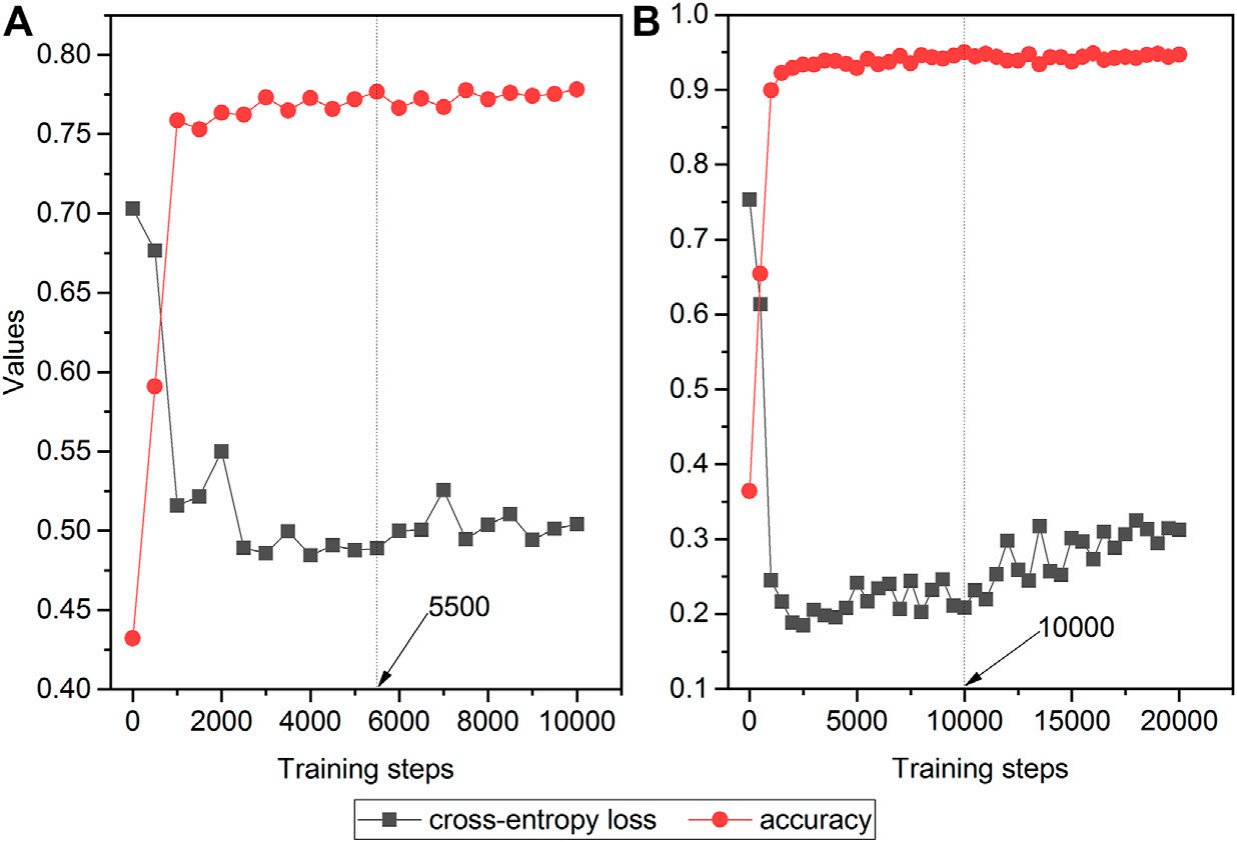
The relationship between cross-entropy loss and accuracy and training steps in fine-tuned ALBERT models: **(A)** Analgesics-induced acute liver failure; and **(B)** Tramadol-related mortalities. The red and gray colors denote the accuracy and cross-entropy loss, respectively.

Figure 3 depicts the trends of loss and accuracy, along with training steps based on development sets. The cross-entropy loss tended to be stable after 4,000 training steps and 5,000 steps for the Analgesics-induced acute liver failure dataset and Tramadol-related mortalities dataset, respectively. Furthermore, the accuracies of the two datasets changed minimally after steps 3,000 and 5,000. Here, we selected the optimized fine-tuned model based on the steps with the maximum accuracy, i.e., 5,500 steps and 10,000 steps for the Analgesics-induced acute liver failure dataset and Tramadol-related mortalities dataset, respectively.

### Causal Inference

To investigate whether the proposed InferBERT approach could capture the causal factors aligned with clinical knowledge, we further carried out the do-calculus analysis to decipher the causal factors for the Analgesics-induced acute liver failure and Tramadol-related mortalities datasets. There are 42 and 48 clinical terms enriched with an adjusted *p* value less than 0.05 using a one-tail z-test for the Analgesics-induced acute liver failure and Tramadol-related mortalities datasets, respectively (see Supplementary Table S1). The clinical terms were distributed into different clinical feature categories, including adverse events, primary suspect drug (psd), age, dose, and gender. Among the enriched clinical terms, the clinical terms with the highest z-score in each clinical feature category were considered as root causes of endpoints (Table 3).

**TABLE 3:** Enriched causal clinical terms by the proposed InferBERT AI model.

Clinical categories	Clinical terms	Z-score	Average of do probabilities	Average of not do probabilities	Adjusted *p* value
Analgesics-induced acute liver failure					
primary suspect drug	APAP	153.92	0.84	0.33	< 1E-16
Age	18–39	36.01	0.54	0.35	< 1E-16
Gender	Female	17.06	0.41	0.35	< 1E-16
Dose	Larger than 100 mg	8.93	0.39	0.35	< 1E-16
Outcome	Death	119.33	0.68	0.30	< 1E-16
Tramadol-related mortalities					
Adversary events	Completed suicide	252.27	1.00	0.28	< 1E-16
Age	40–64	18.33	0.44	0.32	< 1E-16
Gender	Male	3.62	0.37	0.34	0.0001
Dose	Drug abuse	38.77	0.74	0.33	< 1E-16
Primary suspect drug	Hydrocodone bitartrate	23.67	0.91	0.36	< 1E-16

For Analgesics-induced acute liver failure, the enriched root causal factors (z-score) including primary suspect drug^—^APAP (153.92), age^—^18–39 (36.01), gender^—^female (17.06), dose^—^larger than 100 mg (8.93), and outcome^—^death (119.33) were enriched, which is highly consistent with the clinical backgrounds mentioned above. For Tramadol-related mortalities, the enriched root causal factors (z-score) consisted of primary suspect drug^—^Hydrocodone Bitartrate (23.66), age^—^40–64 (18.33), gender–male (3.62), dose^—^drug abuse (38.77), and adverse events^—^Completed suicide (252.27), which is aligned with its clinical background.

**FIGURE 4:**
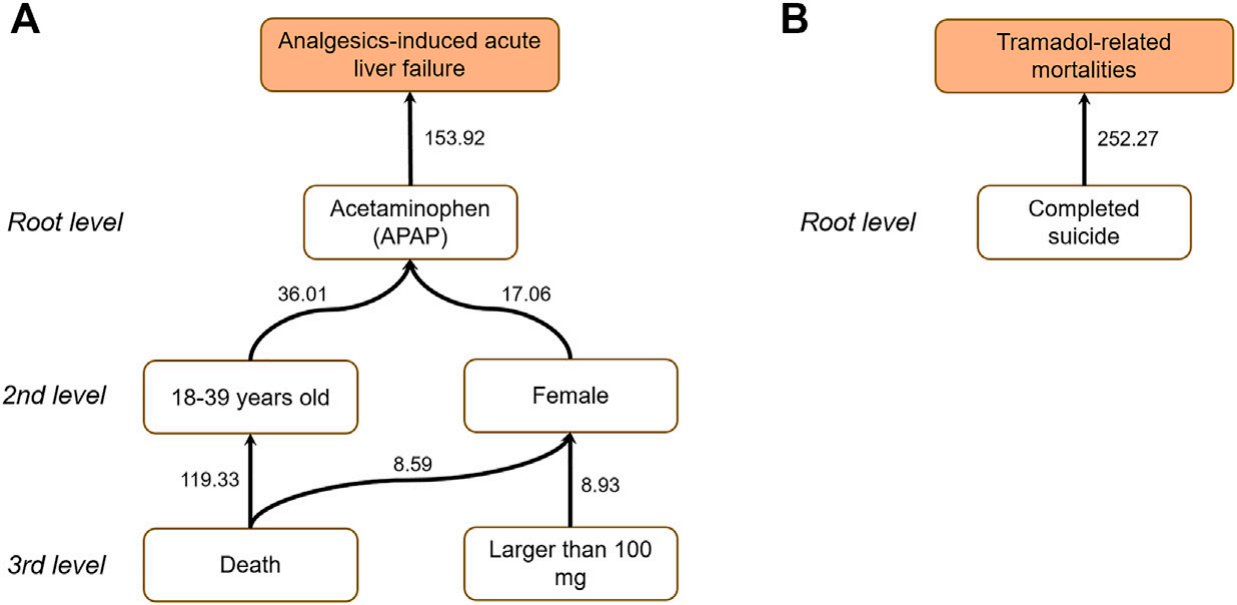
Causal trees for **(A)** Analgesics-induced acute liver failure; and **(B)** Tramadol-related mortalities. The number attached to each arrow denotes the z-score.

To further uncover the interrelationship among causal factors, we implemented a causal tree analysis using the causal factor with highest z-score as a start point. Figure 4 illustrates the constructed causal tree for the endpoints. The link was established with an adjusted *p* value less than 0.05 using a one-tail z-test. For Analgesics-induced acute liver failure, the causal tree penetrated the root cause of Analgesics-induced acute liver failure in patients taking APAP. Furthermore, among the patients taking APAP, the age group 40–64 and women were more likely to take APAP. Moreover, compared to men, women with APAP overdose were more likely to have ALI/ALF, or even death. For Tramadol-related mortalities, the causal tree only consisted of the root level, suggesting that completed suicide was the leading cause of Tramadol-related death.

### Robustness Analysis

**FIGURE 5:**
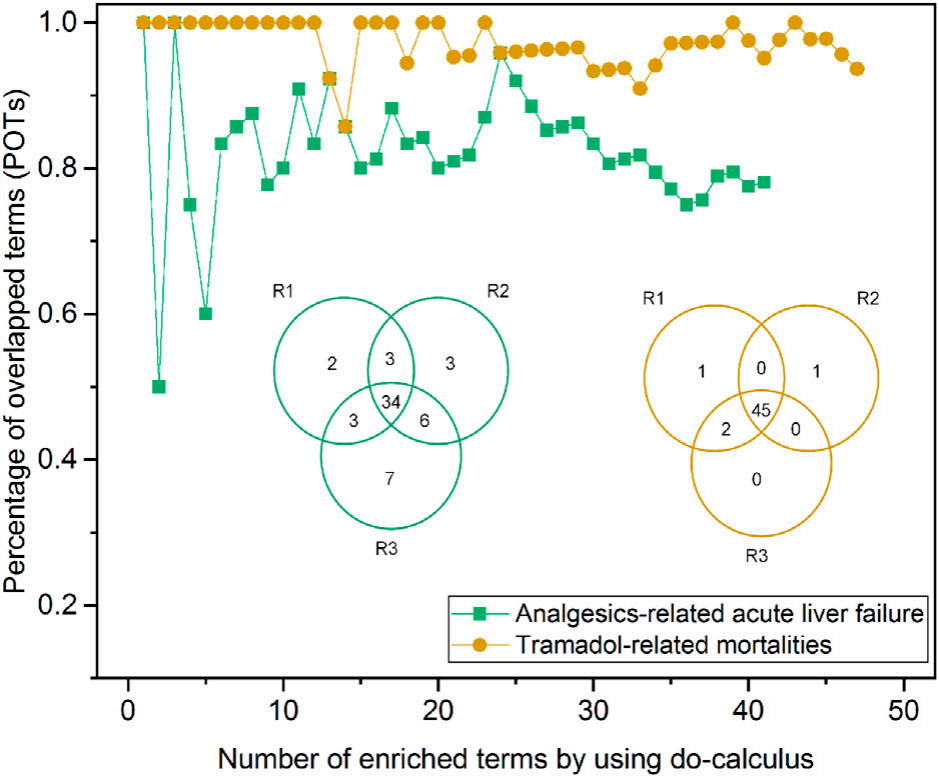
Robustness evaluation of the proposed InferBERT model. The yellow and green colors denote Analgesics-induced acute liver failure and Tramadol-related mortalities datasets, respectively. The Venn diagram illustrates the overlapping of the enriched causal terms by three repeated runs. The percentage of overlapping terms (POPs) shown in the dotted-line curve represent the consistency among ranked order terms from the three repeated runs.

Figure 5 depicts the robustness assessment of the proposed InferBERT model. Venn diagrams showed an average of 82.4 and 95.8% of enriched terms from all the three repeated runs for Analgesics-induced acute liver failure and Tramadol-related mortalities, respectively. Furthermore, the top-ranked enriched terms were very consistent among the top-ranked lists of different repeated runs, as shown in the POT curves of Figure 5. The POT values of the top 10 ranked terms among the three runs were 0.8 and one for Analgesics-induced acute liver failure and Tramadol-related mortalities, respectively. Altogether, the proposed InferBERT model yielded highly repeatable results with great potential for use in further real-world applications. This result indicates that our proposed InferBERT framework is robust, which is an important advantage over other machine learning approaches that are solely based on data without reasoning causality inference.

### Comparison With the Conventional Causal Inference Methods

**FIGURE 6:**
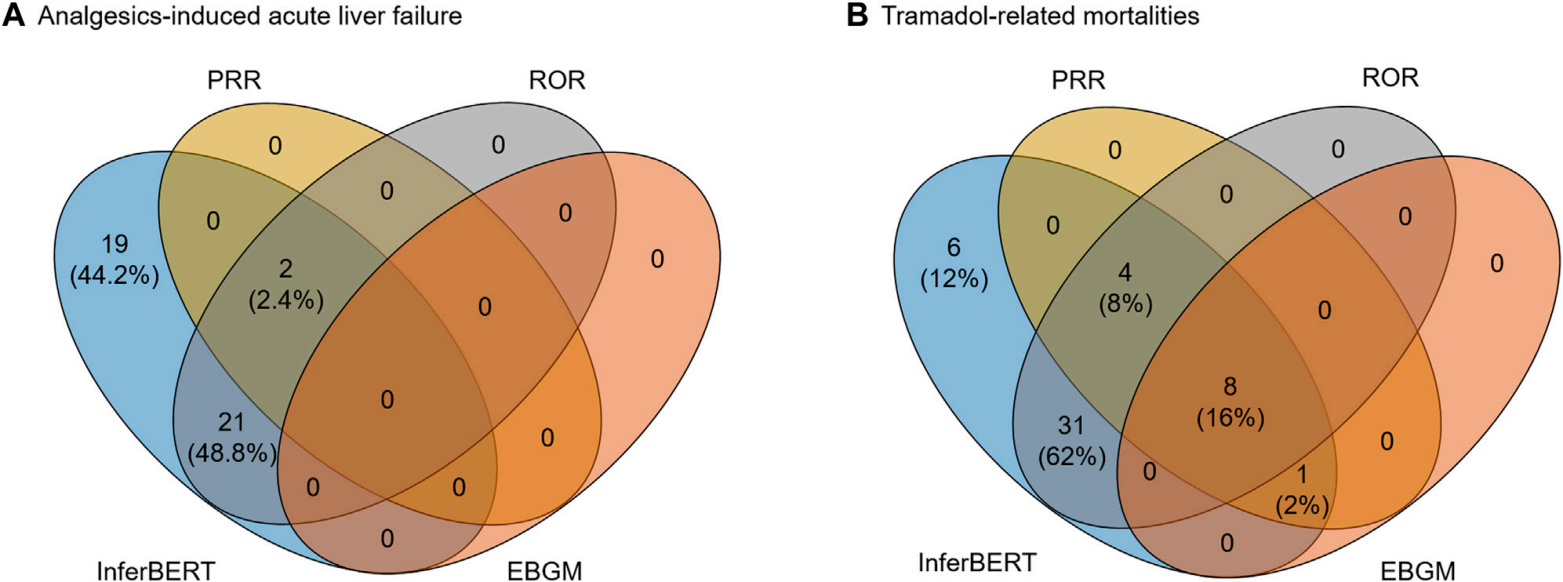
Comparison between the proposed InferBERT model and the three conventional causal inference models including PRR, ROR EBGM: **(A)** Analgesics-induced acute liver failure; and **(B)** Tramadol-related mortalities datasets, respectively.

We further compared the proposed InferBERT model with three conventional signal detection methods (i.e., PRR, ROR, and EBGM) widely applied in pharmacovigilance. Figure 6 illustrated the overlapping terms enriched by the InferBERT model and three conventional methods. The InferBERT model identified more causal factors than three conventional approaches. For Analgesics-induced acute liver failure dataset, the number of enriched terms were ranked as InferBERT model (43 terms) > ROR (23 terms) > PRR (2 terms) > EBGM (0 term). Notably, InferBERT discovers all the causal factors that identified by other conventional methods. On the other hand, all conventional methods missed some of the causal factors discovered by InferBERT, which are verified by the clinical knowledge. Furthermore, the top-ranking terms, (i.e. ranked terms based on the scores in each method) such as “APAP” and “death” were enriched among the four methods, demonstrating the consistency of the proposed method and conventional approaches (Supplementary Table S2). A similar observation was also showed in the Tramadol-related mortalities. The proposed InferBERT model identified the most terms (50), followed by ROR (43), PRR (13), and EBGM (9). The top enriched term “completed suicide” was identified by all four methods. The more enriched terms from the proposed InferBERT model may benefit from the superior ability to uncover the hidden relationship between variables by the transformer model.

## Discussion

Transformer-based language models have greatly expanded the potential of NLP applications. However, few attempts have been made to apply transformer-based language models to address an unmet need for enhanced model-based reasoning for causality. To our best knowledge, the current study and description of InferBERT is the first to succeed in causal inference, aimed at boosting pharmacovigilance. To investigate the performance of our proposed InferBERT model, we used two FAERS case studies, Analgesics-induced acute liver failure and Tramadol-related mortalities, to prove the concept. The root causes of the two datasets were identified, and the results were consistent with the causal relationship derived from real-world data. Moreover, the proposed causal tree seamlessly linked the enriched causal factors into a hierarchical structure to decipher the interrelationship among the causal factors. Furthermore, the high reproducibility of the proposed InferBERT model warrants its potential real-world application.

The FAERS database is an essential resource for hypothesis generation to support pharmacovigilance. However, FAERS data derive from a spontaneous submission by pharmaceutical companies and physicians. There are many data integrity issues such as duplicate records, unstandardized terminologies, missed values, and missing information. Tremendous efforts have been made to clean, normalize, and standardize the data and format, enabling researchers to fully take advantage of the datasets (Banda et al., 2016). In this study, we have used an innovative approach to convert FAERS case reports to sentence-like descriptions as the input for transformer-based language models. This greatly simplified the data preprocessing and overcame the need for a process to handle any missing values. In this study, we employed the preprocessed FARES data curated by the commercial database PharmaPendium (https://www.pharmapendium.com/login), where the original FAERS data is preprocessed for consolidating all relevant data, normalizing different term usage, de-duplicating records, and mapping to either RxNorm (for drugs) or any other controlled terminology (for adverse events), as well as negations. For the further application of the original FAERS, the positive/negative sample definition should be more cautious since the negation issue could deteriorate the quality of positive and negative classification and further decrease the reliability of the causal inference results.

To demonstrate the performance of the proposed InferBERT model, we employed synthetic sentences constructed by standard terminology from the processed FAERS data. The data quality of data resources is crucial for applying the model for causality analysis. For example, the complex causal relationship is embedded in the electronic medical records (EMR), which is essential to suggest the right clinical decision and improve the clinical outcome. Initial efforts such as ClinicalBERT have been proposed to address the clinical questions. A further investigation to combine the ClinicalBERT Huang et al.(2019) with our proposed causal inference strategy may be a promising direction to expand the utility of the current InferBERT model.

There are two limitations in the current version of the InferBERT model, which needs to further investigation. First, we developed the InferBERT model based on FAERS data with a fixed pattern. Further investigation on the different types of free-text data in the biomedical fields is a “must” to evaluate the generalization of the proposed model. Second, we only investigated the model performance with two endpoints (i.e., Analgesics-related acute liver failure and Tramadol-related death). The proposed InferBERT model should be further evaluated with diverse free text-based biomedical datasets to lay out the pros and cons in real-world applications. 

It would be valuable to consider some additional studies to investigate potential further improvement of the proposed InferBERT model. Firstly, the proposed InferBERT model was developed based on the ALBERT_base_ model. Other transformer-based language models could be further investigated to improve causal inference results. A comparative analysis between different transformer models on the improved performance is strongly recommended. The comparison could address the impact factor of model performance such as computational power, computer time, and improvement of model performance, which could be very helpful to select the “fit-for-purpose” model to carry out the causal inference toward real-world application. Secondly, the language model represents the interrelationship of variables in a probabilistic graph. Therefore, Bayesian theory could be considered as a possible route to improve causal inference. The proposed model needs to predefine the endpoint to carry out the causal analysis. The combination of the transformer model and Bayesian approaches may be a promising solution to comprehensively evaluate the causal relationship among different variables in the data. Thirdly, in the current study, we focus on the identification of causal factors of the endpoint. The developed InferBERT model could be utilized to test the potential influence of endpoints for any term combination, which may provide further confidence and establish a causality-based Question and Answering system. Lastly, the current developed InferBERT model is a supervised-based causal inference system. Future work for self-learning of interrelationships among variables directly derived from the pre-trained language models may provide a more intelligent way to identify causal factors for any clinical outcome.

Despite the current attention around AI, most AI-powered language models focus on predicting outcomes rather than understanding causality. Here, we explored the potential utility of transformer-based language models for causal inference in pharmacovigilance. We hope our study can further trigger community interest to examine the potential of AI for understanding the data and to improve the causal interpretability of AI models in the biomedical field.

## Data Availability

The original contributions presented in the study are included in the article/Supplementary Material, further inquiries can be directed to the corresponding authors.
